# A Contracted DNA Repeat in *LHX3* Intron 5 Is Associated with Aberrant Splicing and Pituitary Dwarfism in German Shepherd Dogs

**DOI:** 10.1371/journal.pone.0027940

**Published:** 2011-11-23

**Authors:** Annemarie M. W. Y. Voorbij, Frank G. van Steenbeek, Manon Vos-Loohuis, Ellen E. C. P. Martens, Jeanette M. Hanson-Nilsson, Bernard A. van Oost, Hans S. Kooistra, Peter A. Leegwater

**Affiliations:** 1 Department of Clinical Sciences of Companion Animals, Faculty of Veterinary Medicine, Utrecht University, Utrecht, The Netherlands; 2 Department of Biochemistry, American University of the Caribbean, Cupecoy, St. Maarten, Netherlands Antilles; Temasek Life Sciences Laboratory, Singapore

## Abstract

Dwarfism in German shepherd dogs is due to combined pituitary hormone deficiency of unknown genetic cause. We localized the recessively inherited defect by a genome wide approach to a region on chromosome 9 with a lod score of 9.8. The region contains *LHX3*, which codes for a transcription factor essential for pituitary development. Dwarfs have a deletion of one of six 7 bp repeats in intron 5 of *LHX3*, reducing the intron size to 68 bp. One dwarf was compound heterozygous for the deletion and an insertion of an asparagine residue in the DNA-binding homeodomain of LHX3, suggesting involvement of the gene in the disorder. An exon trapping assay indicated that the shortened intron is not spliced efficiently, probably because it is too small. We applied bisulfite conversion of cytosine to uracil in RNA followed by RT-PCR to analyze the splicing products. The aberrantly spliced RNA molecules resulted from either skipping of exon 5 or retention of intron 5. The same splicing defects were observed in cDNA derived from the pituitary of dwarfs. A survey of similarly mutated introns suggests that there is a minimal distance requirement between the splice donor and branch site of 50 nucleotides. In conclusion, a contraction of a DNA repeat in intron 5 of canine *LHX3* leads to deficient splicing and is associated with pituitary dwarfism.

## Introduction

A defect in the differentiation of endocrine cells of the pituitary gland can lead to isolated or combined pituitary hormone deficiency (CPHD). Normally, the various cell types of the adenohypophysis arise in a distinct temporal order from progenitor cells. The corticotropic cells are the first to differentiate followed by gonadotropes, thyrotropes, lactotropes and somatotropes [1], [2]. Several of the transcription factors involved in the differentiation cascade of the endocrine cells of the pituitary have been identified [3]. In humans and mice, CPHD is mostly related to mutations in genes encoding the transcription factors POU1F1 (previously known as PIT1) and PROP1 [4]–[9]. Mutations in other genes that cause CPHD are rare.

**Figure 1:**
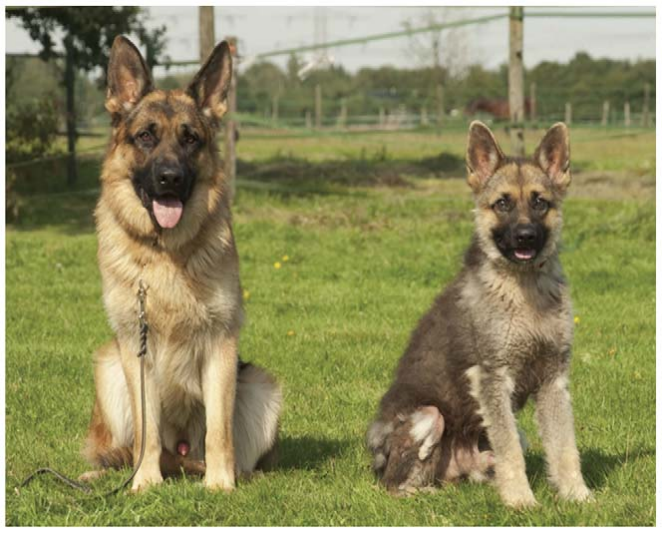
Two fourteen-month-old male German shepherd dogs from the same litter. A healthy German shepherd dog (C8, left) and his littermate that is affected by pituitary dwarfism (C6, right). Note the proportionate growth retardation, the retention of puppy hairs and the lack of guard hairs of the dwarf.

In dogs, congenital growth hormone deficiency or pituitary dwarfism is the most striking form of CPHD. This condition is encountered most often in German shepherd dogs (GSD). Common clinical manifestations are marked growth retardation, retention of secondary hairs (puppy coat) with concurrent lack of primary or guard hairs, and bilateral symmetrical alopecia (Figure 1) [10]. Pituitary dwarfism in the GSD breed is characterized by underdevelopment of the pituitary gland and a combined deficiency of growth hormone, thyroid stimulating hormone, prolactin, and gonadotropins. In contrast, the secretion of adrenocorticotropic hormone is unaffected in these animals [11], [12]. Therefore, the genetic defect that leads to pituitary dwarfism must preclude effective expansion of pituitary stem cells during or after the differentiation of the corticotropic cells. Should the basic defect be known, the disorder would be a model for pituitary dwarfism in man. Involvement of *POU1F1* and *PROP1* in canine CPHD has been excluded before [13], [14]. We set out to locate the causative gene by a genome wide linkage analysis. We found that pituitary dwarfism in GSD is associated with a deletion of a 7 bp repeat in intron 5 of *LHX3*, a gene encoding another important transcription factor involved in pituitary development. It is demonstrated that this contraction of the intron results in deficient splicing.

## Results

### Gene localization

Pituitary dwarfism in GSD is inherited recessively [15]. As is the case with many recessive traits in dog breeds, all dwarfs were expected to be homozygous for the same gene defect due to inbreeding. We started the step-by-step localization of the gene by linkage analysis of two families with in total three dwarfs, using a genome wide set of microsatellite markers (Figure S1A and S1B). There were 49 markers that passed the first selection criterion of homozygosity of dwarfs A7 and A8 for the same allele and sharing of at least one copy of this allele by dwarf B3. Of these markers, 21 were monomorphic in families A and B. Eleven of the remaining markers were heterozygous in only two parents and four markers were heterozygous in three parents. There was only one marker, REN177B24 on CFA09, for which all four parents were heterozygous. The three dwarfs were homozygous for allele 366 of this marker and their analyzed siblings were heterozygous. Therefore, REN177B24 was the first marker to be analyzed further according to our selection criteria and all available dwarfs, parents, and siblings were genotyped with this marker. Three of the 23 dwarfs were not homozygous for the allele of 366 bp (D15, F4, F9). Just one out of nine available parents (D9) and one out of 17 normal siblings (D14) were homozygous for this allele. The homozygous sibling was an offspring of the homozygous parent. The frequency of the associated 366 bp allele was 94% in the group of dwarfs, 56% in the parents, and 44% in the siblings. These results showed that the dwarfism phenotype is strongly associated with marker REN177B24.

To define the critical region, we selected microsatellite markers neighboring REN177B24 from the genomic reference DNA sequence. Markers FH2885, REN256F13, CA1, CA3, CA5, CA7 and CA8 were found to be polymorphic and were subsequently used to genotype the study group. This fine-mapping revealed a genomic region containing three markers, markers CA1 and CA5 at position 51.1 Mb, and marker CA8 at 52.6 Mb of CFA09, for which all dwarfs were homozygous (Figure S1A–S1F). The multipoint lod score for linkage between pituitary dwarfism and the CFA09 region reached a maximum value of 9.8 in the available family material. The markers REN256F13 at position 49.5 Mb and REN177B24 at 54.1 Mb of CFA09 bordered the critical region. Respectively seven (D13, D15-17, E26, E28, F9) and three dwarfs (D15, F4, F9) were heterozygous for these markers and one dwarf was homozygous for another allele of REN256F17 (E27). The region contained 137 genes, according to the annotation of the NCBI, build 2.1 (www.ncbi.nlm.nih.gov/mapview). One of these genes, at position 52.6 Mb of CFA09, encoded LHX3, a transcription factor essential for pituitary gland formation [16], [17]. We considered this gene to be the prime candidate for involvement in GSD dwarfism.

### Mutation screening of *LHX3*

**Figure 2:**
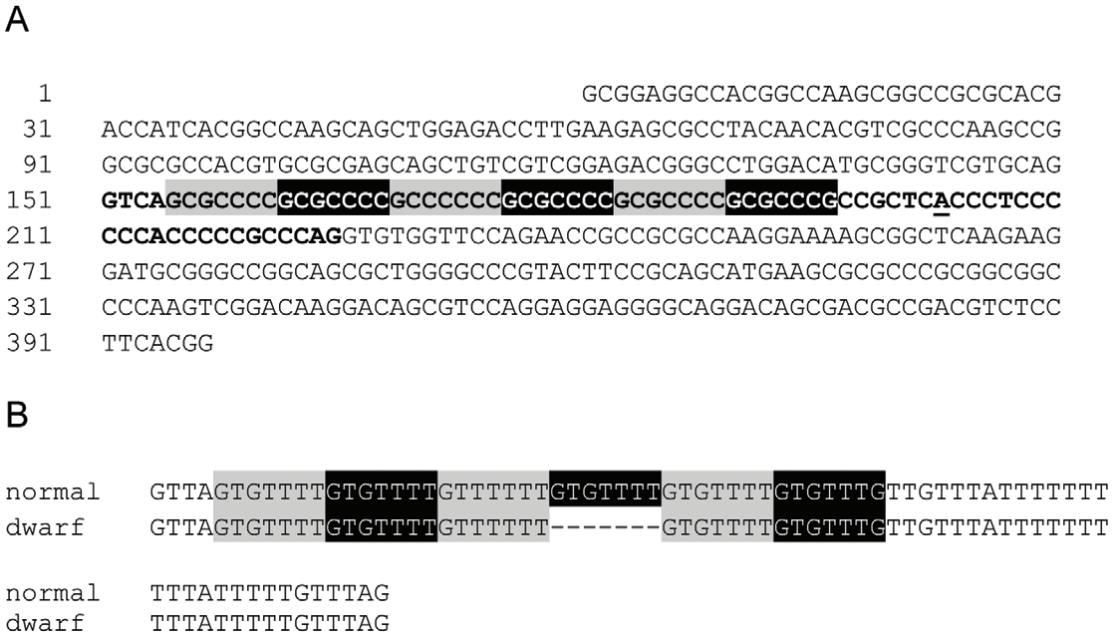
Structure of exon 5, intron 5, and exon 6 of canine *LHX3*. (A) The DNA sequence derived from BAC clone RP81-17A4 fills a gap in the reference genome sequence build 2.1. The intron is indicated by bold characters and the putative splice branch site is underlined. The intron contained 6 repeats of the 7 bp sequence GCGCCCC, indicated by alternating grey and black blocks. The G at position 3 of the third repeat was mutated to a C and the last repeat ends with a G-residue. The Genbank accession number of the DNA sequence is Q913875. (B) Comparison of the DNA sequence of intron 5 in the normal German shepherd dogs B4 and C8 and the dwarfs B3 and C6 after bisulfite treatment of genomic DNA. The treatment converts unmethylated cytosine residues to uracil residues, which act like thymine in the PCR. The intron in the normal dogs B4 and C8 (Fig. S2) consisted of 75 bp and contained 6 repeats of the 7 bp sequence which are converted to GTGTTTT, indicated by alternating grey and black blocks. Seven consecutive nucleotides in the region of repeats 4–6 are deleted from the intron of the dwarfs B3 and C6, indicated by dashes (NM_001197187, c.622-37-31del).

The gene structure of canine *LHX3* was deduced from BLAST comparisons between human cDNA sequences and the genome of the dog. The intron 5-exon 6 junction was not covered by build 2.1 of the reference DNA sequence. The gap was filled by DNA sequence analysis of BAC clone RP81-17A4 that carries canine *LHX3* (Figure 2A). We analyzed the DNA sequence of the 7 exons and the intron boundaries in 4 dwarfs (B3, A8, D13, E22) and did not detect mutations in comparison to the amended version of the gene. We noted that intron 5 consists of only 75 bp, is very GC-rich, and contains 6 imperfect repeats of a 7 bp sequence. Expansions of DNA repeats of up to 12 bp are potentially pathogenic [18], [19]. We therefore wanted to establish whether the dwarfism phenotype was associated with expansion of the 7 bp repeat region in intron 5. We found that DNA fragments containing the GC-rich intron cannot be PCR-amplified with standard thermostable DNA polymerases. These fragments could however be amplified after bisulfite conversion of cytosine residues to uracil [18]. In addition, these fragments could be amplified with the use of Platinum pfx DNA polymerase [20]. Surprisingly, the DNA fragments from dwarfs that contain *LHX3* intron 5 were shorter than the fragments from control dogs. The DNA sequence showed that the size difference is the result of a deletion of one 7 bp repeat unit from the intron (Figure 2B). All dwarfs except one (F9), displayed homozygosity for the 7 bp deletion, all available parents were heterozygous carriers and their siblings were either carrier or did not have the 7 bp deletion. These relatives included the 3 parents and 4 siblings that were homozygous like the dwarfs for allele 243 of marker CA8. This result established that *LHX3* was located within the linked region with zero recombination. Dwarf F9, which was not homozygous for the deletion, was a compound heterozygote with a second mutation described below. Seven dogs from a group of 37 unrelated GSD from the Dutch population with normal growth were carrier of the 7 bp deletion (allele frequency = 0.094).

### *In vitro* splicing assay

**Figure 3:**
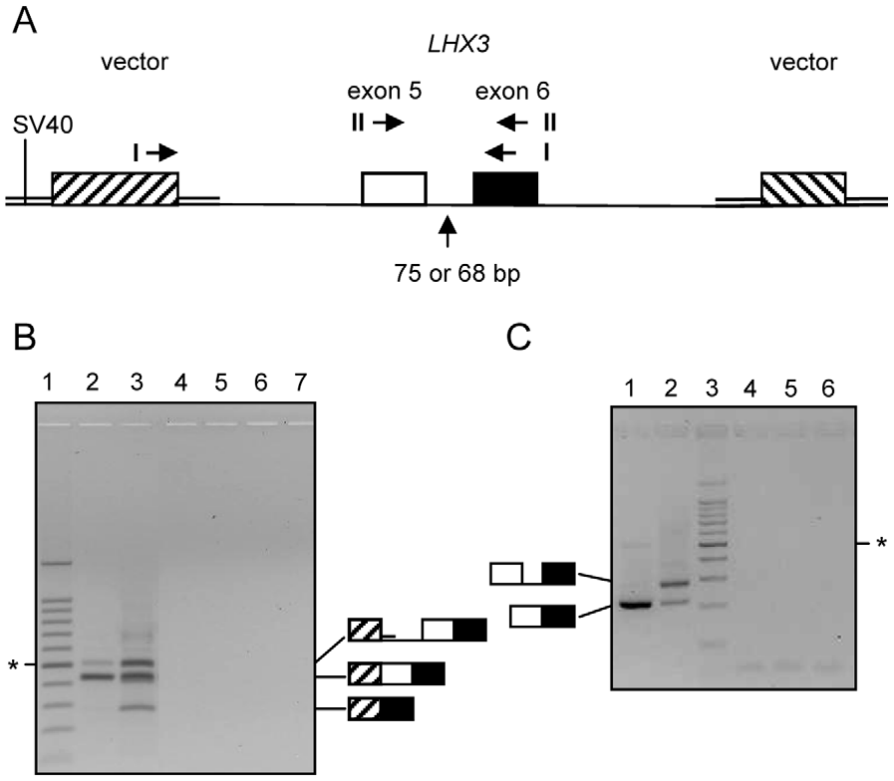
Defective splicing due to a deletion in intron 5 of canine *LHX3* demonstrated by exon trapping. (A) Map of part of the plasmids used for transfection of COS-7 cells. The open and closed boxes represent exons 5 and 6 of canine *LHX3*, as indicated. The hatched boxes represent chimeric exons that consist of HIV-1 *tat* and rabbit *HBB2* gene sequences. The double lines indicate vector sequences of pSPL3b [50]. The recombinant DNA is transcribed from the SV40 promoter at the indicated position. The map is not drawn to scale. The horizontal arrows indicate the positions of the PCR primer sets I and II that were used on cDNA derived from the cells. Primer set II was adapted to bisulfite treatment of the RNA. The vertical arrow points out intron 5, consisting of 75 bp in the control and 68 bp in the mutant construct. (B,C) Eukaryotic COS-7 cells were transfected with recombinant plasmids containing the control or the mutant intron 5 of canine *LHX3*. After 2 days, RNA was isolated and analyzed by RT-PCR. The PCR products were analyzed by agarose gel electrophoresis in the presence of ethidium-bromide. The structure of the fragments was obtained by DNA sequence analysis of bands excised from the gels. These structures are indicated by drawings as in (A). (B) The 7 bp deletion leads to exon 5 skipping in a proportion of the transcripts. The products were analyzed with primer set I. Lane 1: size marker = 100 bp ladder, * indicates the 500 bp fragment; lane 2: products derived from cells transfected with plasmids containing intron 5 of 75 bp; lane 3: intron 5 of 68 bp; lanes 4 and 5: as lanes 2 and 3, respectively, except that reverse transcriptase was omitted to exclude contamination of RNA substrate with plasmid DNA; lane 6: transfection with vector pSPL3b without insert; lane 7: no transfection. Primer set I does not amplify fragments containing intron 5 due to the high cytosine content of the intron. (C) The 7 bp deletion leads to retention of intron 5 in a proportion of the transcripts. The RNA was treated with bisulfite and analyzed with primer set II. Lanes 1 and 4: products derived from cells transfected with plasmids containing intron 5 of 75 bp; lanes 2 and 5: products derived from cells transfected with plasmids containing intron 5 of 68 bp; lane 6: transfection with vector pSPL3b without insert; lane 3: size marker = 100 bp ladder, * indicates the 500 bp fragment. Lanes 4 and 5: omission of reverse transcriptase to exclude contamination of RNA substrate with plasmid DNA.

We supposed that the mutant size of *LHX3* intron 5 of 68 nucleotides (nt) could be too small to be spliced efficiently. We tested this possibility by applying an exon trapping assay. Cultured eukaryotic cells were transfected with recombinant plasmids that carried multiple introns and exons under the control of a promoter of transcription. The splicing machinery of the host cell processed the transcript if the proper signals were present and the RNA products could be characterized by RT-PCR. A pSPL3b-derived plasmid was constructed that included exon 5, intron 5 and exon 6 of *LHX3* derived from BAC RP81-17A4. This construct was used to create a second plasmid with a 7 bp deletion in intron 5 identical to the mutation in intron 5 of GSD dwarfs (Figure 3A).

COS-7 cells were transfected with either construct or mock transfected and the transcripts were analyzed using RT-PCR with primer set I that spanned the first vector derived exon and exon 6. The products included the fragment expected from normal splicing (Figure 3B, lanes 2 and 3). In addition, a larger product was seen with both constructs in which the chimeric intron was retained. The identity of the products was confirmed by DNA sequence analysis. A product of approximately 280 bp was apparent with the mutant construct but hardly detectable with the construct containing *LHX3* intron 5 of normal size. This fragment was shown by sequence analysis to consist of the first recombinant exon spliced to exon 6 of *LHX3*, so it represented skipping of exon 5 in the splicing process.

Another result that could be expected from a defective intron would be retention of that intron. A technical problem to detect retention of intron 5 was posed by the inherent difficulty of this intron to be amplified by PCR because of the high cytosine content. Analogous to the resolution of strong base pairing in DNA, strong base pairing in the cDNA was prevented by treatment of the RNA with bisulfite prior to cDNA synthesis. The plasmids described above that contained mutant or normal intron 5 were used again, but in this experiment the products were analyzed by RT-PCR primer set II that spanned intron 5 only and had sequences adapted to the bisulfite conversion. Cells transfected with the plasmid containing the normal intron produced spliced RNA of the anticipated size (Figure 3C, lane 1). The same product and a larger, more prominent product were found in cells transfected with the mutant construct (Figure 3C, lane 2). The larger product was derived from RNA that had retained the mutant intron 5. Again, the identity of the fragments was established by DNA sequence analysis of the excised bands. These results are consistent with our supposition that the mutant intron 5 of 68 nt is defective.

### *LHX3* cDNA analysis

**Figure 4:**
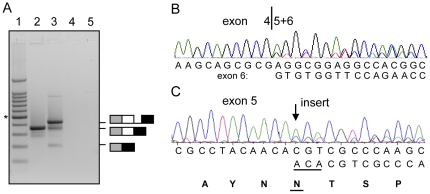
Splicing products of *LHX3* RNA in German shepherd dwarfs. (A) Normal splicing of intron 5 as well as retention of the intron was observed in the pituitary of one of the dwarfs (F8). The cDNA fragments were generated with Platinum pfx polymerase and primers LHX3 ex4-7 F and LHX3 intron5 R in exons 4 and 6, respectively (Table S4). The fragments were analyzed by agarose gel electrophoresis. Lane 1: 100 bp size standard, * indicates the 500 bp fragment; lane 2: fragment derived from a control dog of mixed breed without the 7 bp deletion in intron 5 or other *LHX3* mutations; lane 3: fragments derived from a dwarf homozygous for the deletion in intron 5; lanes 4 and 5: as lanes 2 and 3, respectively, without reverse transcriptase. The structure of the fragments as indicated by the drawings was confirmed by DNA sequence analysis of the excised bands. Open box: exon 5, closed box: exon 6, line: intron 5. (B) The cDNA sequence of the same dwarf, obtained with primers in exons 4 and 7 and standard taq DNA polymerase, indicates that exon 5 was skipped in a proportion of the transcripts. (C) The other dwarf (F9) for which pituitary RNA was available displayed heterozygosity for an insertion of an ACA trinucleotide sequence (underlined) in exon 5. This insertion translates into insertion of an asparagine residue (underlined) in the amino acid sequence (bold).

To establish whether the splice defects observed with the exon trapping assay could be detected *in vivo*, we analyzed *LHX3* cDNA from the available pituitary glands of two dwarfs (F8 and F9) and of a normal dog of mixed breed. In addition to normally spliced products, dwarf cDNA displayed retention of intron 5 in a proportion of the transcripts with a processed intron 4, and skipping of exon 5 in another part of the products (Figure 4A). These splicing products were not seen in the cDNA from the normal dog. Skipping of exon 5 of 152 nt results in a frame shift (NP_001184116.1, p.E159fs). The translation product would lack the highly conserved, DNA-binding homeodomain. Retention of the mutant intron of 68 nt leads to an insertion of 21 amino acids and a frameshift at the protein level (p.V208ins21fs). The first triplets of both intron 5 and exon 6 are valine codons.

Splicing defects are known to induce nonsense-mediated decay of the RNA [21] and the intron 5 mutation could lead to a low RNA expression level. Quantitative RT-PCR experiments indicated that the expression of *LHX3* in the homozygous dwarf F8 was approximately 100-fold lower than in a normal dog.

Remarkably, one of the two dwarfs (F9) of which the cDNA was analyzed, showed to be heterozygous for an insertion of an ACA trinucleotide sequence (Figure 4C). The insertion occurred at a site of two ACA triplets that are normally present in exon 5 (NM_001197187, c.545_547dupACA). The result for the open reading frame was an insertion of an AAC codon for asparagine at position 182 of the LHX3b protein isoform (p.N182dup), which is located in the first α-helix of the homeodomain of LHX3. The triplet insertion and the heterozygosity of the dwarf were confirmed by sequence analysis of exon 5 from genomic DNA (Figure S2A). The insertion is situated close to intron 5 and can be amplified as part of the same fragment. The heterozygous dwarf displayed the fragment with the 7 bp deletion in addition to a fragment that was 3 bp longer than the wild type fragment due to the triplet insertion (Figure S2B). Exon 5 of the other dwarfs was analyzed but none of these had the allele with the triplet insertion. No other mutations were identified in the complete *LHX3* cDNA from the dwarfs, as expected from the genomic DNA sequence analysis. The analysis of the complete cDNA corrected the Genbank annotation of the gene and will be described elsewhere.

## Discussion

Pituitary dwarfism in German shepherd dogs has been seen for decades and dwarfs are born in purebred populations all over the world. Genome wide linkage analysis with microsatellite markers was used to localize the genetic defect causing this disorder. The stepwise approach was similar to that described by Leegwater et al. [22]. In the first step, a limited number of DNA samples from two informative families were typed with all markers. Candidate regions were selected and ranked using loose criteria and analyzed one by one in all available dogs. Fortunately, the marker REN177B24 on chromosome 9 that ranked highest after the first step turned out to be associated with dwarfism in the study group. The followed strategy saved time and reduced expenses in comparison with a screen of the marker set in the complete study group.

The linkage of the REN177B24 region with the phenotype was confirmed by analysis of closely spaced markers. The multipoint lod score of 9.8 for linkage between the region on CFA09 and the dwarfism phenotype indicated that the mutated gene is located in this region. The critical region contained one gene of major interest, i.e., *LHX3* (also known as *LIM-3* or *P-Lim*). LHX3 is a member of the LIM homeodomain protein family of DNA-binding transcription factors. These factors regulate the expression of genes that pattern the body and are critical for cell specialization during embryonic development [23]. Molecular defects in the *LHX3* gene are associated with the CPHD syndrome in humans. Ten different homozygous *LHX3* defects have been reported in 24 human patients from consanguineous families [24]–[28]. Most human patients display a complete deficit of all anterior pituitary hormones, except for adrenocorticotropic hormone. In mice, *LHX3* is essential for differentiation and proliferation of pituitary cell lineages [16]. Homozygous *LHX3*-knockout mice are stillborn or die within 24 h of birth. Such mice display a complete absence of the differentiated hormone secreting cells, except for some corticotropes [16], [17]. Because the endocrinological phenotype of humans with *LHX3* mutations and *LHX3*-knockout mice is consistent with the phenotype of the GSD dwarfs, we considered *LHX3* an excellent candidate gene for involvement in pituitary dwarfism in this breed.

Our results show that mutations in the *LHX3* gene are associated with pituitary dwarfism of GSD. Analysis of intron 5 revealed a 7 bp deletion that was associated with defective splicing of a proportion of the transcripts *in vivo* and *in vitro*. The aberrant splicing products resulted from skipping of exon 5 or retention of intron 5. Skipping of exon 5 results in a frame shift; the translation product will lack the homeodomain and will therefore probably not be functional [29]. Retention of the mutant intron of 68 nt also leads to a frame shift in the part of the mRNA that codes for the homeodomain. The splice donor, acceptor and putative branch sites are not altered by the mutation and the deleted sequence GCGCCCC is not similar to any known intronic splice enhancer. Therefore, the splicing of the mutant intron 5 must be hampered by a structural constraint such as its size.

Natural deletion mutants and *in vitro* experiments indicate that there is a minimum size of 65–78 nt of introns in higher eukaryotes [30]–[38]. It appears that a universal threshold of minimal intron size cannot be defined (Table 1). In the case of murine *notch4* an intron of 68 nt is spliced normally, while a mutant intron of 71 nt of human *PKD1* with apparently normal splicing signals is deleterious [33], [35]. It seems plausible that demands on intron size are dictated by requirements on spacing between the donor and the branch site and between the branch site and the splice acceptor [39]–[41]. The first reaction of splicing is a nucleophilic attack of the branch site on the 5′ splice donor, resulting in lariat formation. The loop of the lariat is composed of the fragment between the 5′ donor and branch site. From known deletion mutants and *in vitro* experiments it can be concluded that splicing is hampered when this distance drops below 50 nt (Table 1). Like for total size, this threshold is not strict since a distance of 50 nt is normal in mouse *notch4* but deleterious in human *RECQL4*. The overlap between normal and shortened donor-branch site distances is slightly less than the overlap between the sizes of complete and mutant introns, suggesting that the first parameter better defines the size requirement. There is no structural constraint to form an RNA lariat with a loop of 50 nt. Steric hindrance of splicing factors probably impedes splicing if the distance between the splice donor and branch site is too small [39]. The deletion in canine *LHX3* shortened the distance between the splice donor and branch site to 48 nt. It did not affect the RNA sequence context of intron 5 because the deleted sequence is one of the 6 direct tandem repeats normally present in the intron. Therefore we can reliably conclude that steric parameters retard the splicing of intron 5.

**Table 1:** Distance parameters of normal and defective introns.

		Intron	Intron size (nt)		Donor-branch distance (nt)		
Gene	Gene ID	number	normal	mutant	normal	mutant	Reference
*PKD1*	5310	43	75	57	52	34	32
*PKD1*	5310	43	75	55	52	32	32
*PKD1*	5310	31	90	71	67	48	33
*RECQL4*	9401	8	77	66	61	50	34
*notch4*	18132	10	68	60	50	42	35
*GNPTG*	84572	8	76	43	?	?	38
*SLC34A3*	142680	9	167	66	145	44	36
*SLC34A3*	142680	10	139	54	77	-	36
*SLC4A11*	610206	7	87	68	63	42	37
recombinant			81	47	59	47	30
AdC *E1A*	2652980		114	78	86	49	39
*LHX3*	607584	5	75	68	53	46	this study

? = no clear branch site.

- = no branch site.

The size of intron 5 of *LHX3* of mammals ranges from 75 nt in dog and horse to 114 nt in cow (Table S1). The GC content of the intron is invariably high, but the analyzed species other than dog lack repetitive elements, reducing the chance of size alterations during DNA replication. Therefore the risk of size reduction of the intron, affecting expression of the gene, is probably low for these species.

The splicing deficiency of the canine *LHX3* intronic deletion in the *in vitro* system was not absolute and accordingly we detected wild type *LHX3* mRNA in the two available pituitaries from dwarfs. Possible variations in the level of residual activity between dwarfs could be related to the high level of phenotypic variability that we observe [10]. Because there was only one pituitary available of a dwarf that was homozygous for the 7 bp deletion, we could not adequately evaluate the expression level of *LHX3* in dwarfs. The qPCR experiment of the one sample indicated that the *LHX3* mRNA level is strongly reduced. This reduction is less obvious from the comparison of the cDNA products of a dwarf and a normal dog in Figure 4A. It should be noted however that this experiment was not quantitative and only shows the endpoint of the PCR. Similarly, the intensity of the two bands in lane 2 of Figure 4A cannot be used for an quantitative assessment of the level of intron retention because the fragment with the retained intron is probably subject to nonsense mediated decay leading to underrepresentation of the corresponding band.

Due to the high GC content of intron 5, we experienced difficulties to amplify this intron by PCR. Bisulfite treatment, used to convert cytosine bases to uracil, is known to resolve strong base-pairing and allow the analysis of GC-rich DNA sequences. We applied it in this study to modify cytosine rich RNA before cDNA synthesis. To the best of our knowledge, this is the first report of the application of bisulfite treatment of RNA to allow RT-PCR.

One of the dwarfs turned out to be a compound heterozygote for an insertion of an ACA triplet in exon 5 and the repeat deletion in intron 5 of *LHX3*. The insertion leads to duplication of an asparagine residue in the first α-helix of the DNA-binding homeodomain of the protein. Although in a number of vertebrates the LHX3 protein does have two consecutive N-residues at the position of this mutation, the insertion is still expected to demolish the function of the homeodomain. This is because the spacing of amino acids that contact the DNA binding site is critical and the size of the domain is highly conserved [42]. A blast search of the non-redundant protein database with human LHX3 shows that the length of the homeodomain of all known vertebrate LHX1, LHX2, LHX3 and LHX4 proteins is identical.

Fragment length analysis showed that the two mutations occurred independently, i.e., that the triplet insertion was not a secondary mutation in an allele of the *LHX3* gene that was already dysfunctional because of the 7 bp deletion. Apparently by coincidence, this dwarf was homozygous for the same three markers as all other dwarfs (Figure S1F). The detection of two mutations in *LHX3* supports our conclusion that this gene is involved in the dwarfism phenotype in GSD, since the odds that we would have detected two mutations in a strong positional candidate gene by chance are negligible. The high frequency of 0.094 of the deletion allele in a group of Dutch GSDs is not abnormal in pet genetics [43]. It warrants the implementation of a DNA test to prevent mating of carriers and reduce the spread of the disorder in the breed.

In this study, we have demonstrated that a contracted DNA repeat in intron 5 of *LHX3* could well be responsible for CPHD in GSD by leading to aberrant splicing. Pituitary dwarfism in GSD forms a natural model for experimental therapies of CPHD in humans. One important requirement, identification of the associated gene, has now been fulfilled.

## Materials and Methods

### Ethics Statement

The dogs entered in this study, presented to the University Clinic for Companion Animals in Utrecht for diagnosis, treatment and sometimes subsequent euthanasia for compassionate reasons, were examined and handled by a licensed veterinarian (HSK). Pituitary function tests were performed and blood samples were collected in the course of routine diagnosis and care. All dogs were privately owned and included with informed consent of the owners. Thus we complied to the conditions set forth in the Dutch ‘Wet op de Uitoefening van de Diergeneeskunde’ (Law on the Practice of Veterinary Medicine) of March 21, 1990 and approval of an ethics committee for the use of samples of the animals was not necessary.

### Animals

The study group comprised 23 GSD with pituitary dwarfism, 9 of their healthy parents, 17 of their healthy siblings, 37 unrelated GSD with normal growth and 1 healthy control dog of mixed breed. There were two sibling pairs and one sibling trio of dwarfs in the group of patients (Figure S1A, S1D and S1E). Three parents produced dwarfs with different mates (Figure S1E). The diagnosis of pituitary dwarfism was based on clinical manifestations and the results of a combined anterior pituitary simulation test using four releasing hormones, i.e., growth hormone-releasing hormone, thyrotropin-releasing hormone, gonadotropin-releasing hormone, and corticotropin-releasing hormone, according to methods described previously [12], [44], [45]. Blood samples were collected and genomic DNA was isolated from the samples by a salt extraction method [46]. The origin and the birth year of each dwarf and available related dogs is listed in Table S2).

### Linkage analysis

A genome wide linkage analysis was performed with two families of GSD in which three dwarfs were born, i.e., pedigrees A and B (Figure S1A and S1B). The dwarfs (A7, A8, B3), parents (A5, A6, B1, B2), and two healthy siblings (A9, A10) were genotyped with 256 microsatellite markers listed in Table S3. The microsatellite markers were selected from the 5000cR hybrid radiation map and from the marker set derived thereof (Guyon et al. 2003). The criteria for candidate regions in the genomic DNA were that the dwarfs from pedigree A had to be homozygous for the same allele of at least one marker from that region and that the dwarf of pedigree B had to share at least one copy of this allele. The candidate regions were ranked according to the information content of the markers, indicated by the number of heterozygous parents. The highest ranked regions were then further investigated by typing the complete study group with the corresponding markers. To fine-map the candidate regions, additional microsatellites listed in Table S4 were selected from the reference canine DNA sequence using Tandem Repeats Finder [47], after masking of LINE and SINE elements with Repeatmasker (Smit et al. 1996).

The microsatellite markers were genotyped using standard PCR techniques with oligonucleotides of which one was fluorescently labeled at the 5′-end with 6-FAM, TET, HEX, VIC, NED or PET. The products were mixed with size standard 500GS TAMRA or 500GS LIZ of Applied Biosystems (ABI) depending on the oligonucleotide label and analyzed with a 3100 Genetic Analyzer (ABI), using Genescan 3.1 software (ABI) for genotype assessment.

Families A-E and data of markers CA7, REN256F13, CA5, CA1, CA8, REN177B24, CA3 and FH2885 were used for multipoint lod score calculation with Genehunter 2.1 software [48]. We assumed a genetic distance of 1 cM per Mbp and the observed alleles of each marker were assumed to have equal frequencies. The calculation was based on a recessive inheritance model with full penetrance and no phenocopies.

### Characterization of *LHX3*

The RP81 BAC library was screened with a 1.1 kb *Pst*I fragment from mouse *LHX3* cDNA as described previously [49]. The DNA of BAC 17A4 was isolated with the Hispeed plasmid isolation kit (Qiagen). The intron 5-exon 6 junction was sequenced directly from the BAC DNA with primers BAC17A4 F and BAC17A4 R (Table S4) using BigDye v.3.1 and analyzed with an ABI 3100 Genetic Analyzer, according to the protocols of the supplier.

The exons and intron-exon junctions of *LHX3* were amplified from genomic DNA of dwarfs and control dogs using standard PCR techniques with the primers detailed in Table S4.

Genomic DNA that served as a template for PCR amplification of intron 5 of canine *LHX3* was treated with bisulfite and purified with the EZ DNA methylation-gold kit (Zymo Research). The DNA (100 ng) was mixed with buffer and bisulfite, denatured at 98°C for 10 min and incubated at 64°C for 2.5 h. The treated DNA was purified as prescribed by the manufacturer. The PCR reaction was set up to amplify the DNA molecule derived from the strand with the coding sequences. The used primers were BS LHX3 ex5 F and BS LHX3 ex6 R1 (Table S4). In case of fragment analysis, the forward primer was labeled with 6-FAM (Eurogentec) and the products were analyzed with the Genetic Analyzer 3130xl and Genemapper 4.0 software, both from ABI. In case of DNA sequence analysis, the PCR was performed using standard protocols. The PCR products were purified with Shrimp Alkaline Phosphatase (Promega) and Exonuclease I (New England BioLabs), and used as a template in a DNA sequencing reaction containing BigDye Terminator v3.1 (ABI), according to manufacturer's instructions. The reaction products were purified with multiscreen 96-well sephadex 50-gel filtration plates, and analyzed on an ABI 3100 Genetic Analyzer (ABI).

The cDNA of *LHX3* from two dwarfs (F8 and F9) and a control dog of mixed breed was analyzed. Total RNA was obtained from the anterior pituitary using the RNeasy mini kit (Qiagen) and treated with DNaseI as recommended by the manufacturer. The cDNA was synthesized with AMV Reverse Transcriptase (Promega), using oligo-dT as primer. Overlapping fragments were amplified with the primer pairs described in Table S4.

For the quantification of *LHX3* mRNA, cDNA was synthesized with the Iscript kit (Bio-Rad). Quantitative PCR was performed with the IQ SYBR Green supermix (Bio-Rad) with primers LHX3 ex3-4 F, which overlapped exons 3 and 4, and LHX3 ex4 R (Table S4) in exon 4 of *LHX3*, all according to the manufacturer's protocols. The annealing temperature was 68°C. The gene *RPS5* was used as a reference with primers RPS5 F and RPS5 R (Table S4) and the data was analyzed with MyiQ iCycler (Bio-Rad). The RNA that was used for the qPCR experiment was isolated from the anterior pituitary of the male dwarf F8, euthanized at an age of 11 months and from the normal male dog of mixed breed at the age of 4 years.

The pituitary cDNA fragments that spanned intron 5 were amplified with Platinum pfx DNA polymerase (Invitrogen) with primers LHX3 intron5 F and LHX3 intron5 R (Table S4). The hybridization temperature of the PCR was 64°C and the elongation temperature was 68°C.

The DNA sequences were aligned using the Seqman program from Lasergene software (DNASTAR) and compared to the reference dog genome with BLAST software (www.ncbi.nlm.nih.gov/BLAST). The Seqman program was also used to align the cDNA sequences obtained from the dwarfs with those obtained from the healthy control dog.

### Splicing assay

The normal intron 5 of canine *LHX3* was subcloned with the neighboring exons 5 and 6 as a *Not*I-*Bam*HI fragment of 1040 bp from the canine BAC 17A4 in the plasmid pSPL3b. The *Not*I site is located 43 bp upstream of exon 5 and the *Bam*HI site is 597 bp downstream of exon 6. The cloning site of pSPL3b is situated in the HIV-1 *tat* intron flanked by the proper *tat* exon-intron junctions. The transcription of the recombinant exons and introns is under control of a SV40 promoter [50]. A mutant intron with the deletion of the GSD dwarfs was constructed with the recombinant plasmid as a PCR template, a phosphorylated oligonucleotide of 82 nt spanning the mutant intron and a phosphorylated oligonucleotide positioned adjacent to the first primer in the opposite strand. The high fidelity Phusion polymerase was used for this purpose under conditions recommended by the supplier (New England Biolabs). The PCR products were circularized with T4 DNA ligase and used to transfect competent JM109 bacteria (Promega). The presence of the mutation was confirmed by DNA sequence analysis.

The simian COS-7 cell line (6×10^4^ cells) was transfected using Lipofectamin 2000, according to the protocol of the manufacturer (Invitrogen). After growth for two days, the RNA was isolated from the cells with the RNeasy Mini Kit (Qiagen) and treated two times with DNase I. The iScript kit (Bio-Rad) was used to produce cDNA with random primers. The RNA (500 ng) was modified with bisulfite and purified in the same manner as DNA, except that the initial denaturation step was 1 min at 98°C, followed by incubation at 64°C for 30 min. The iScript kit (Bio-Rad) was used to produce cDNA with random primers. The PCR primers to amplify the region extending from the first, vector derived exon to exon 6 of *LHX3* without prior bisulfite treatment of the RNA were pSPL3b F en LHX3 ex6 R (Fig. 3A, primer set I; Table S4). The PCR primers to amplify the exon 5 – exon 6 region after bisulfite modification were BS LHX3 ex5 F and BS LHX3 ex6 R2 (Fig. 3A, primer set II; Table S4). In case of single reaction products, the DNA fragment was sequenced directly as described above. In case of multiple products, the bands were separated by electrophoresis on an agarose gel, excised, purified with the Qiaquick gel extraction kit (Qiagen), and sequenced as described above.

The positions of the putative branch site of normal and defective introns from the literature were derived by comparison of the intron with a published U2-dependent branch site consensus sequence [51].

## Supporting Information

Figure S1 **Cosegregation of pituitary dwarfism with a region of canine chromosome CFA09 in German shepherd dwarfs.** (A,B) A genome wide analysis was performed with microsatellite markers of Table S1 and DNA of the dwarfs of litters A and B, their parents and siblings A9 and A10. The genotypes of marker REN177B24 were consistent with the presence of the gene for dwarfism. Analysis of closely situated markers confirmed the homozygosity by descent of the region in the dwarfs. (C–E) The region was analyzed in other available families confirming the cosegregation with dwarfism. (F) Nine isolated cases of pituitary dwarfism also displayed homozygosity in the region. The region between REN256F13 and REN177B24 was identically homozygous in all dwarfs. The dwarf indicated F9 turned out to be a compound heterozygote for two independent mutations. The alleles associated with pituitary dwarfism are highlighted in green.
(PDF)

Figure S2 **Demonstration of DNA mutations in genomic DNA fragments of** ***LHX3*** **in German shepherd dwarfs.** (A) Partial DNA sequence of exon 5 in dwarf F9 confirms heterozygosity of a trinucleotide insertion (underlined) observed in cDNA from the same dog. (B–D) The site of the insertion and intron 5 are amplified together by PCR with a 6-FAM labeled primer. (B) The normal German shepherd dog B4 displays a single fragment of 240 bp. (C) All dwarfs except F9 are homozygous for the 7 bp deletion in intron 5 as shown by a single fragment of 233 bp as for dwarf B3. (D) The dwarf F9 displays two alleles with either the deletion or the insertion of 3 bp.
(PPT)

Table S1 **Size and DNA sequence of intron 5 of LHX3 in mammals.**
(XLS)

Table S2 **Birth year, sex and origin of German shepherd dogs included in the study of pituitary dwarfism.**
(XLS)

Table S3 **Genome wide set of microsatellite markers.**
(DOC)

Table S4 **DNA sequences of oligonucleotides used in the study of pituitary dwarfism.**
(XLS)
