# Supplementary material for: A Contracted DNA Repeat in LHX3 Intron 5 Is Associated with Aberrant Splicing and Pituitary Dwarfism in German Shepherd Dogs
Source: PLoS One. 2011 Nov 23;6(11):e27940. doi: 10.1371/journal.pone.0027940 (PMC3223203; doi:10.1371/journal.pone.0027940)
Supplement: Figure S1 — Cosegregation of pituitary dwarfism with a region of canine chromosome CFA09 in German shepherd dwarfs. (A,B) A genome wide analysis was performed with microsatellite markers of Table S1 and DNA of the dwarfs of litters A and B, their parents and siblings A9 and A10. The genotypes of marker REN177B24 were consistent with the presence of the gene for dwarfism. Analysis of closely situated markers confirmed the homozygosity by descent of the region in the dwarfs. (C–E) The region was analyzed in other available families confirming the cosegregation with dwarfism. (F) Nine isolated cases of pituitary dwarfism also displayed homozygosity in the region. The region between REN256F13 and REN177B24 was identically homozygous in all dwarfs. The dwarf indicated F9 turned out to be a compound heterozygote for two independent mutations. The alleles associated with pituitary dwarfism are highlighted in green. (PDF) [file pone.0027940.s001.pdf]

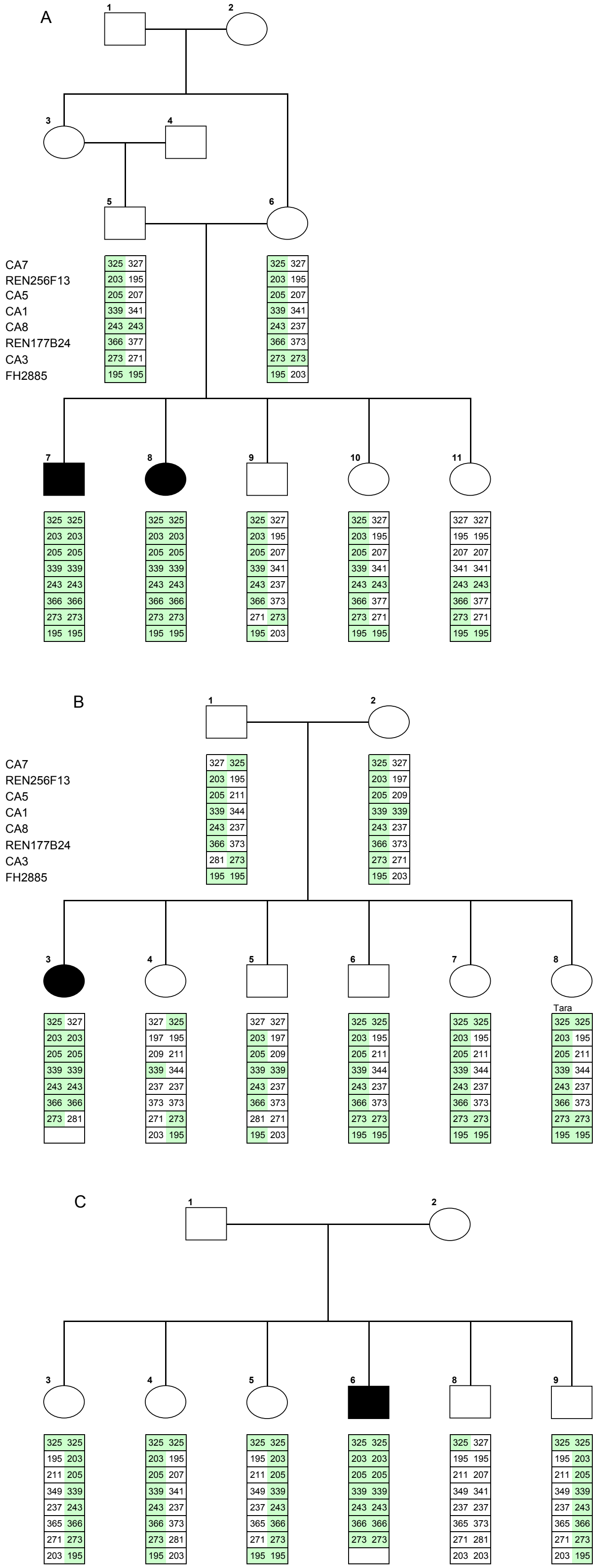
A
CA7
REN256F13
CA5
CA1
CA8
REN177B24
CA3
FH2885
1
2
3
4
5: 325 327; 203 195; 205 207; 339 341; 243 243; 366 377; 273 271; 195 195
6: 325 327; 203 195; 205 207; 339 341; 243 237; 366 373; 273 273; 195 203
7: 325 325; 203 203; 205 205; 339 339; 243 243; 366 366; 273 273; 195 195
8: 325 325; 203 203; 205 205; 339 339; 243 243; 366 366; 273 273; 195 195
9: 325 327; 203 195; 205 207; 339 341; 243 237; 366 373; 271 273; 195 203
10: 325 327; 203 195; 205 207; 339 341; 243 243; 366 377; 273 271; 195 195
11: 327 327; 195 195; 207 207; 341 341; 243 243; 366 377; 273 271; 195 195
B
CA7
REN256F13
CA5
CA1
CA8
REN177B24
CA3
FH2885
1: 327 325; 203 195; 205 211; 339 344; 243 237; 366 373; 281 273; 195 195
2: 325 327; 203 197; 205 209; 339 339; 243 237; 366 373; 273 271; 195 203
3: 325 327; 203 203; 205 205; 339 339; 243 243; 366 366; 273 281
4: 327 325; 197 195; 209 211; 339 344; 237 237; 373 373; 271 273; 203 195
5: 327 327; 203 197; 205 209; 339 339; 243 237; 366 373; 281 271; 195 203
6: 325 325; 203 195; 205 211; 339 344; 243 237; 366 373; 273 273; 195 195
7: 325 325; 203 195; 205 211; 339 344; 243 237; 366 373; 273 273; 195 195
8
Tara: 325 325; 203 195; 205 211; 339 344; 243 237; 366 373; 273 273; 195 195
C
1
2
3: 325 325; 195 203; 211 205; 349 339; 237 243; 365 366; 271 273; 203 195
4: 325 325; 203 195; 205 207; 339 341; 243 237; 366 373; 273 281; 195 203
5: 325 325; 195 203; 211 205; 349 339; 237 243; 365 366; 271 273; 195 195
6: 325 325; 203 203; 205 205; 339 339; 243 243; 366 366; 273 273
8: 325 327; 195 195; 211 207; 349 341; 237 237; 365 373; 271 281; 203 203
9: 325 325; 195 203; 211 205; 349 339; 237 243; 365 366; 271 273; 203 195

D

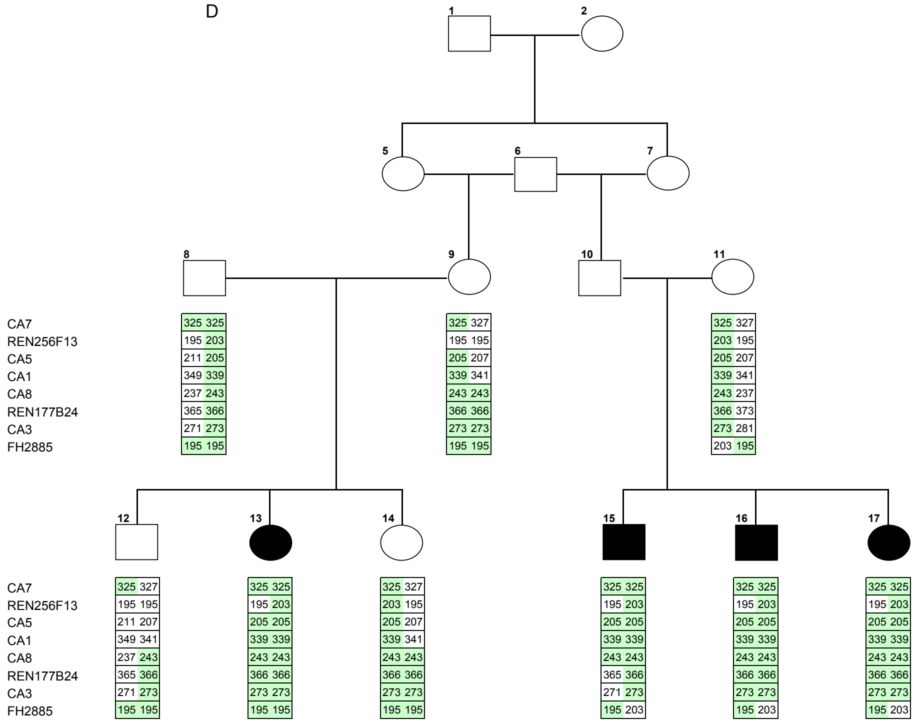
1
2
5
6
7
8
9
10
11
CA7
REN256F13
CA5
CA1
CA8
REN177B24
CA3
FH2885
8: 325 325 / 195 203 / 211 205 / 349 339 / 237 243 / 365 366 / 271 273 / 195 195
9: 325 327 / 195 195 / 205 207 / 339 341 / 243 243 / 366 366 / 273 273 / 195 195
11: 325 327 / 203 195 / 205 207 / 339 341 / 243 237 / 366 373 / 273 281 / 203 195
12: 325 327 / 195 195 / 211 207 / 349 341 / 237 243 / 365 366 / 271 273 / 195 195
13: 325 325 / 195 203 / 205 205 / 339 339 / 243 243 / 366 366 / 273 273 / 195 195
14: 325 327 / 203 195 / 205 207 / 339 341 / 243 243 / 366 366 / 273 273 / 195 195
15: 325 325 / 195 203 / 205 205 / 339 339 / 243 243 / 365 366 / 271 273 / 195 203
16: 325 325 / 195 203 / 205 205 / 339 339 / 243 243 / 366 366 / 273 273 / 195 203
17: 325 325 / 195 203 / 205 205 / 339 339 / 243 243 / 366 366 / 273 273 / 195 203

E

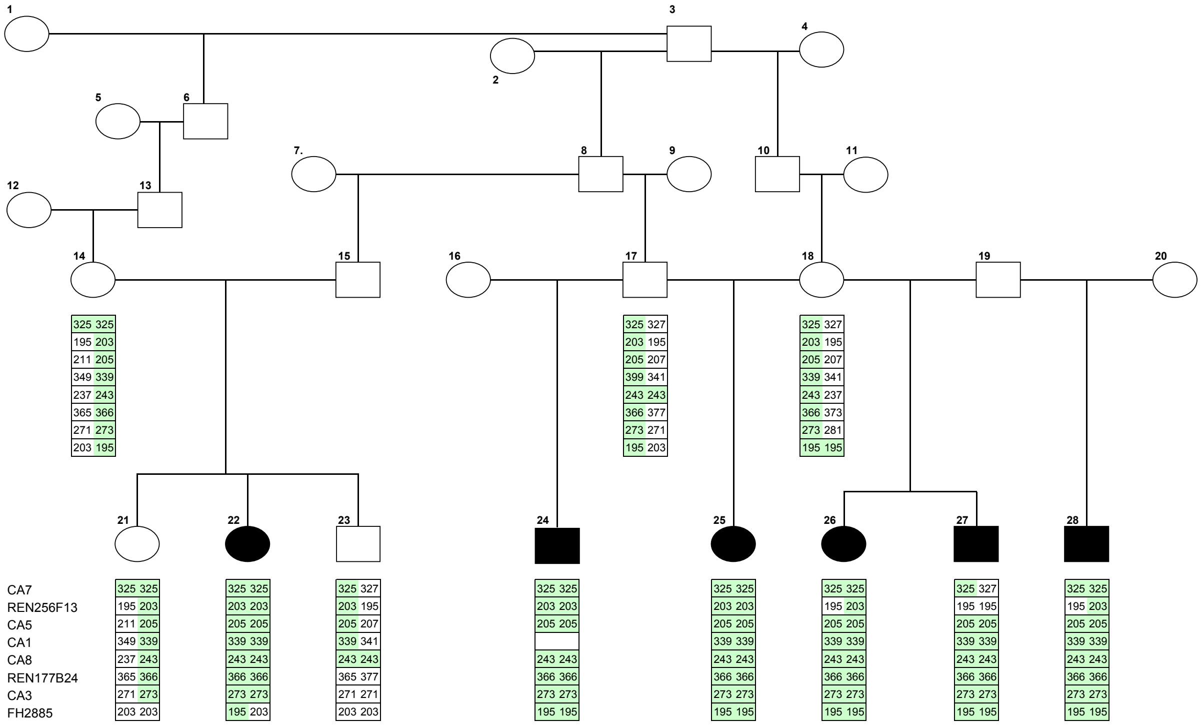
1
2
3
4
5
6
7.
8
9
10
11
12
13
15
16
19
20
CA7
REN256F13
CA5
CA1
CA8
REN177B24
CA3
FH2885
14: 325 325 / 195 203 / 211 205 / 349 339 / 237 243 / 365 366 / 271 273 / 203 195
17: 325 327 / 203 195 / 205 207 / 399 341 / 243 243 / 366 377 / 273 271 / 195 203
18: 325 327 / 203 195 / 205 207 / 339 341 / 243 237 / 366 373 / 273 281 / 195 195
21: 325 325 / 195 203 / 211 205 / 349 339 / 237 243 / 365 366 / 271 273 / 203 203
22: 325 325 / 203 203 / 205 205 / 339 339 / 243 243 / 366 366 / 273 273 / 195 203
23: 325 327 / 203 195 / 205 207 / 339 341 / 243 243 / 365 377 / 271 271 / 203 203
24: 325 325 / 203 203 / 205 205 / / 243 243 / 366 366 / 273 273 / 195 195
25: 325 325 / 203 203 / 205 205 / 339 339 / 243 243 / 366 366 / 273 273 / 195 195
26: 325 325 / 195 203 / 205 205 / 339 339 / 243 243 / 366 366 / 273 273 / 195 195
27: 325 327 / 195 195 / 205 205 / 339 339 / 243 243 / 366 366 / 273 273 / 195 195
28: 325 325 / 195 203 / 205 205 / 339 339 / 243 243 / 366 366 / 273 273 / 195 195

F

	1	2	3	4	5	6	7	8	9
CA7	325 325	325 325	325 325	325 325	325 325	333 333	325 325	325 325	325 327
REN256F13	203 203	203 203	203 203	203 203	203 203	203 203	203 203	203 203	203 195
CA5	205 205	205 205	205 205	205 205	205 205	205 205	205 205	205 205	205 205
CA1	339 339	339 339	339 339	339 339	339 339	339 339	339 339	339 339	339 339
CA8	243 243	243 243	243 243	243 243	243 243	243 243	243 243	243 243	243 243
REN177B24	366 366	366 366	366 366	366 377	366 366	366 366	366 366	366 366	366 373
CA3	273 273	273 273	273 273	273 271	273 273	273 273	273 281	273 281	273 281
FH2885	195 195	195 203	195 195	195 197	195 195	203 203			
