# Supplementary figures and images for: A Contracted DNA Repeat in LHX3 Intron 5 Is Associated with Aberrant Splicing and Pituitary Dwarfism in German Shepherd Dogs
Source: PLoS One. 2011 Nov 23;6(11):e27940. doi: 10.1371/journal.pone.0027940 (PMC3223203; doi:10.1371/journal.pone.0027940)

## Slide 1
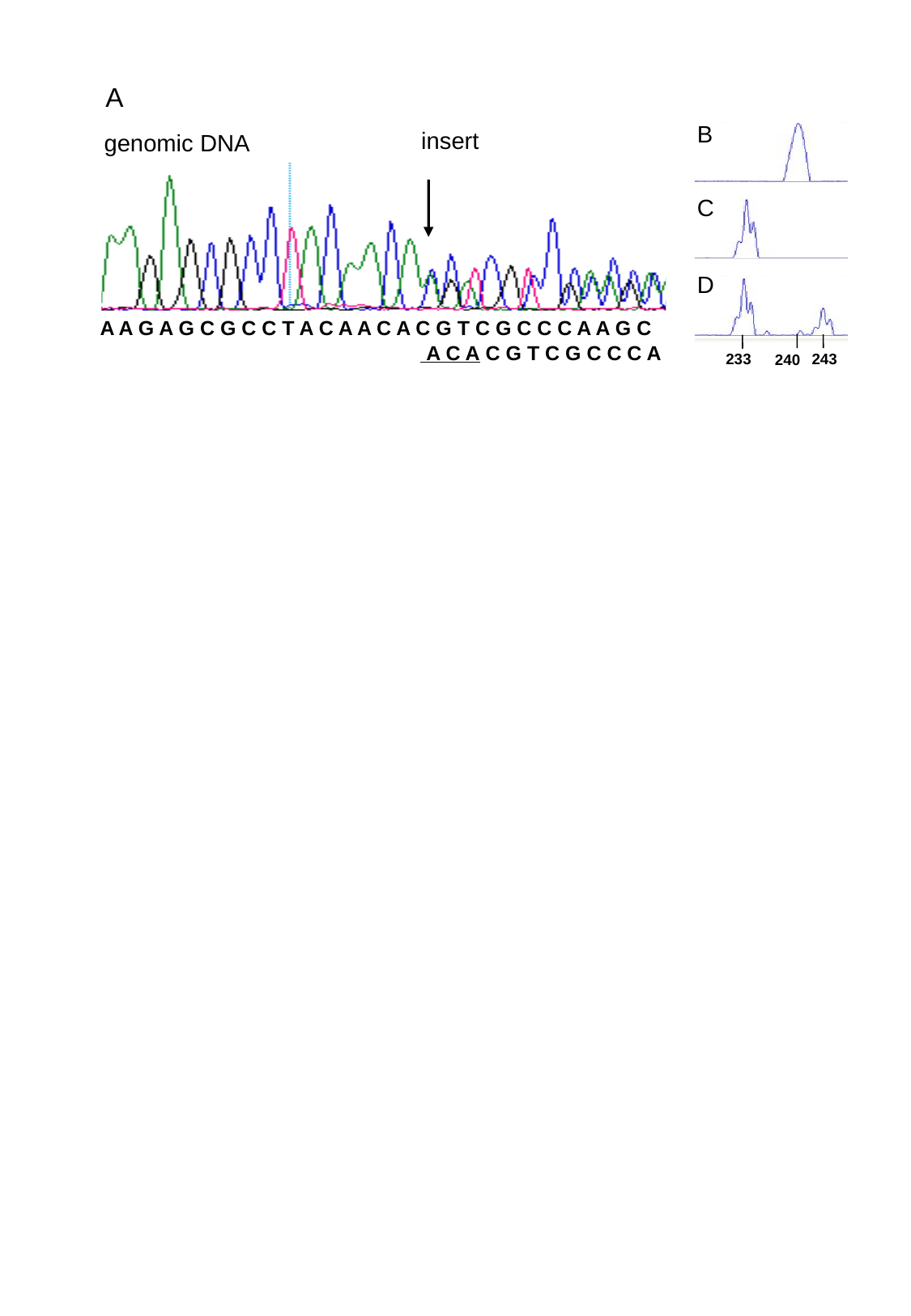

A
B
insert
genomic DNA
C
D
 A A G A G C G C C T A C A A C A C G T C G C C C A A G C
 A C A C G T C G C C C A
233
243
240

Supplement: Figure S2 — Demonstration of DNA mutations in genomic DNA fragments of LHX3 in German shepherd dwarfs. (A) Partial DNA sequence of exon 5 in dwarf F9 confirms heterozygosity of a trinucleotide insertion (underlined) observed in cDNA from the same dog. (B–D) The site of the insertion and intron 5 are amplified together by PCR with a 6-FAM labeled primer. (B) The normal German shepherd dog B4 displays a single fragment of 240 bp. (C) All dwarfs except F9 are homozygous for the 7 bp deletion in intron 5 as shown by a single fragment of 233 bp as for dwarf B3. (D) The dwarf F9 displays two alleles with either the deletion or the insertion of 3 bp. (PPT) [file pone.0027940.s002.ppt]
