# Supplementary material for: A Contracted DNA Repeat in LHX3 Intron 5 Is Associated with Aberrant Splicing and Pituitary Dwarfism in German Shepherd Dogs
Source: PLoS One. 2011 Nov 23;6(11):e27940. doi: 10.1371/journal.pone.0027940 (PMC3223203; doi:10.1371/journal.pone.0027940)
Supplement: Table S3 — Genome wide set of microsatellite markers. (DOC) [file pone.0027940.s005.doc]

| **Table S3. Genome wide set of microsatellite markers** | |  |  |  |  |  |
| --- | --- | --- | --- | --- | --- | --- |
| Marker | CFA | Mb |  | Marker | CFA | Mb |
| FH2452 | 1 | 10 |  | FH2525 | 6 | 8 |
| FH2016 | 1 | 11 |  | FH2576 | 6 | 10 |
| FH2313 | 1 | 20 |  | AHT109 | 6 | 20 |
| FH3413 | 1 | 25 |  | REN149M14 | 6 | 30 |
| REN162B09 | 1 | 37 |  | FH2119 | 6 | 47 |
| REN122I01 | 1 | 48 |  | C06.636 | 6 | 60 |
| C01.424 | 1 | 59 |  | FH3972 | 7 | 12 |
| C01.251 | 1 | 64 |  | FH2917 | 7 | 18 |
| FH2309 | 1 | 85 |  | REN162C04 | 7 | 29 |
| AHT138 | 1 | 97 |  | REN286O18 | 7 | 33 |
| FH2326 | 1 | 108 |  | FH2379 | 7 | 44 |
| FH2294 | 1 | 115 |  | REN200G14 | 7 | 50 |
| REN149E24 | 2 | 10 |  | FH2201 | 7 | 62 |
| FH2087U | 2 | 11 |  | FH2581 | 7 | 70 |
| REN150M24 | 2 | 36 |  | FH2301 | 7 | 80 |
| FH2237 | 2 | 50 |  | FH2973 | 7 | 81 |
| FH2608 | 2 | 63 |  | FH3241 | 8 | 4 |
| C02.894 | 2 | 70 |  | FH3425 | 8 | 14 |
| AHT111 | 2 | 76 |  | REN67O13 | 8 | 19 |
| REN161A12 | 3 | 13 |  | C08.410 | 8 | 33 |
| FH2137 | 3 | 14 |  | C08.373 | 8 | 45 |
| FH2131 | 3 | 30 |  | FH2149 | 8 | 62 |
| FH2320 | 3 | 45 |  | FH2138 | 8 | 65 |
| REN92B17 | 3 | 50 |  | C08.618 | 8 | 68 |
| FH3464 | 3 | 57 |  | GALK | 9 | 8 |
| REN260I04 | 3 | 68 |  | C09.250 | 9 | 12 |
| FH2316 | 3 | 70 |  | FH2263 | 9 | 16 |
| REN216N05 | 3 | 77 |  | C09.173 | 9 | 19 |
| FH2107 | 3 | 84 |  | C02.342 | 9 | 21 |
| FH2302 | 3 | 92 |  | REN01E05 | 9 | 25 |
| REN298N18 | 4 | 5 |  | FH2186 | 9 | 35 |
| FH2773 | 4 | 9 |  | REN278L10 | 9 | 48 |
| REN171H02 | 4 | 15 |  | REN177B24 | 9 | 54 |
| FH3310 | 4 | 20 |  | FH2885 | 9 | 60 |
| FH2412 | 4 | 40 |  | REN287G01 | 9 | 63 |
| FH2142 | 4 | 51 |  | C10.404 | 10 | 3 |
| REN160J02 | 4 | 70 |  | FH4081 | 10 | 12 |
| AHT103 | 4 | 75 |  | C10.781 | 10 | 16 |
| FH2457 | 4 | 88 |  | RVC8 | 10 | 23 |
| FH3928 | 5 | 9 |  | FH2293 | 10 | 32 |
| FH2594 | 5 | 10 |  | REN181G20 | 10 | 39 |
| FH2140 | 5 | 13 |  | ZUBECA1 | 10 | 49 |
| REN42N13 | 5 | 16 |  | FH2422 | 10 | 56 |
| ZUBECA6 | 5 | 22 |  | C10.602 | 10 | 66 |
| FH3702 | 5 | 32 |  | FH3381 | 10 | 69 |
| DTR05.8 | 5 | 39 |  | C10.865 | 10 | 87 |
| REN262G24 | 5 | 49 |  |  |  |  |
| REN192M20 | 5 | 51 |  |  |  |  |
| FH2383 | 5 | 66 |  |  |  |  |
| FH3450 | 5 | 76 |  |  |  |  |
| REN122J03 | 5 | 82 |  |  |  |  |
| CPH14 | 5 | 90 |  |  |  |  |
|  |  |  |  |  |  |  |
|  |  |  |  |  |  |  |
| Marker | CFA | Mb |  | Marker | CFA | Mb |
| AHT137 | 11 | 6 |  | DTRCN1 | 17 | 9 |
| FH3203 | 11 | 9 |  | FH2321 | 17 | 12 |
| FH4031 | 11 | 12 |  | REN294E18 | 17 | 27 |
| REN242K04 | 11 | 19 |  | FH4023 | 17 | 40 |
| FH2004 | 11 | 32 |  | FH3995 | 17 | 46 |
| REN245N06 | 11 | 39 |  | PEZ8 | 17 | 63 |
| FH3393 | 11 | 47 |  | FH2869 | 17 | 67 |
| REN194N17 | 11 | 56 |  | FH4060 | 18 | 4 |
| C11.20 | 11 | 65 |  | FH3944 | 18 | 8 |
| C11.873 | 11 | 68 |  | REN186N13 | 18 | 11 |
| DGN13 | 11 | 73 |  | REN54P11 | 18 | 21 |
| C11.750 | 11 | 77 |  | FH3815 | 18 | 33 |
| FH2200 | 12 | 4 |  | FH3824 | 18 | 40 |
| REN258L11 | 12 | 10 |  | REN47J11 | 18 | 45 |
| FH2152 | 12 | 14 |  | FH2429 | 18 | 54 |
| REN213F01 | 12 | 17 |  | AHT130 | 18 | 58 |
| FH3711 | 12 | 24 |  | FH3969 | 19 | 4 |
| FH2401 | 12 | 28 |  | FH2206 | 19 | 18 |
| REN208M20 | 12 | 44 |  | FH3313 | 19 | 25 |
| FH2707 | 12 | 51 |  | FH3491 | 19 | 36 |
| FH2347 | 12 | 63 |  | REN213G21 | 19 | 46 |
| C12.852 | 12 | 74 |  | FH3299 | 19 | 54 |
| C13.391 | 13 | 4 |  | PEZ19 | 20 | 4 |
| FH3494 | 13 | 6 |  | REN55P21 | 20 | 9 |
| REN307K04 | 13 | 15 |  | FH2951 | 20 | 17 |
| FH3619 | 13 | 23 |  | REN100J13 | 20 | 26 |
| REN286P03 | 13 | 33 |  | REN93E07 | 20 | 39 |
| FH2348 | 13 | 37 |  | FH2312 | 21 | 3 |
| REN227M12 | 13 | 45 |  | FH3624 | 21 | 10 |
| AHT121 | 13 | 52 |  | FH3823 | 21 | 20 |
| C13.758 | 13 | 55 |  | FH3880 | 21 | 44 |
| FH3951 | 14 | 10 |  | FH3803 | 21 | 51 |
| C14.866 | 14 | 16 |  | REN49F22 | 22 | 4 |
| FH3725 | 14 | 25 |  | REN128H16 | 22 | 7 |
| FH2658 | 14 | 37 |  | FH3355 | 22 | 13 |
| REN289L09 | 14 | 43 |  | FH3853 | 22 | 64 |
| FH2763 | 14 | 48 |  | FH2508 | 23 | 6 |
| PEZ10 | 14 | 59 |  | FH3078 | 23 | 12 |
| FH3802 | 15 | 4 |  | FH2626 | 23 | 19 |
| FH4012 | 15 | 14 |  | REN181K04 | 23 | 55 |
| REN06C11 | 15 | 21 |  | FH3750 | 24 | 4 |
| FH2535 | 15 | 31 |  | FH3023 | 24 | 7 |
| FH2295 | 15 | 43 |  | FH2159 | 24 | 12 |
| REN193M22 | 15 | 46 |  | REN209L11 | 24 | 18 |
| FH2278 | 15 | 58 |  | FH2495 | 24 | 24 |
| FH3939 | 15 | 67 |  | FH3083 | 24 | 34 |
| REN214L11 | 16 | 6 |  | FH3287 | 24 | 45 |
| FH2670 | 16 | 7 |  | FH3977 | 25 | 6 |
| REN85N14 | 16 | 22 |  | FH2324 | 25 | 17 |
| AHT131 | 16 | 22 |  | FH3979 | 25 | 28 |
| FH2175 | 16 | 35 |  | FH3627 | 25 | 42 |
| REN275L19 | 16 | 42 |  | FH4027 | 25 | 49 |
| FH2155 | 16 | 54 |  | REN299M21 | 26 | 20 |
| FH3592 | 16 | 60 |  | DGN10 | 26 | 26 |
|  |  |  |  |  |  |  |
| Marker | CFA | Mb |  |  |  |  |
| FH4001 | 27 | 10 |  |  |  |  |
| REN277O05 | 27 | 18 |  |  |  |  |
| FH2925 | 27 | 27 |  |  |  |  |
| REN56C20 | 27 | 39 |  |  |  |  |
| FH4019 | 27 | 42 |  |  |  |  |
| REN181L14 | 27 | 46 |  |  |  |  |
| REN72K15 | 27 | 48 |  |  |  |  |
| FH2759 | 28 | 4 |  |  |  |  |
| FH3033 | 28 | 24 |  |  |  |  |
| FH3033 | 28 | 24 |  |  |  |  |
| REN51I12 | 28 | 37 |  |  |  |  |
| FH2952 | 29 | 6 |  |  |  |  |
| FH3878 | 29 | 15 |  |  |  |  |
| REN165M10 | 29 | 20 |  |  |  |  |
| REN164F23 | 29 | 28 |  |  |  |  |
| FH3489 | 30 | 6 |  |  |  |  |
| REN51CV16 | 30 | 12 |  |  |  |  |
| REN248F14 | 30 | 22 |  |  |  |  |
| FH3632 | 30 | 33 |  |  |  |  |
| FH2305 | 30 | 36 |  |  |  |  |
| FH3053 | 30 | 37 |  |  |  |  |
| REN43H24 | 31 | 10 |  |  |  |  |
| FH2582 | 31 | 14 |  |  |  |  |
| FH2239 | 31 | 28 |  |  |  |  |
| REN110K04 | 31 | 30 |  |  |  |  |
| FH2712 | 31 | 38 |  |  |  |  |
| FH2189 | 31 | 52 |  |  |  |  |
| FH2875 | 32 | 15 |  |  |  |  |
| FH3635 | 32 | 20 |  |  |  |  |
| AHT127 | 32 | 26 |  |  |  |  |
| FH3294 | 32 | 29 |  |  |  |  |
| FH2965 | 33 | 9 |  |  |  |  |
| FH3608 | 33 | 13 |  |  |  |  |
| REN291M20 | 33 | 27 |  |  |  |  |
| FH2165 | 33 | 33 |  |  |  |  |
| FH3721 | 34 | 5 |  |  |  |  |
| REN64E19 | 34 | 20 |  |  |  |  |
| REN243O23 | 34 | 27 |  |  |  |  |
| FH3836 | 34 | 35 |  |  |  |  |
| FH4010 | 34 | 43 |  |  |  |  |
| REN125M11 | 35 | 12 |  |  |  |  |
| REN282I22 | 35 | 19 |  |  |  |  |
| FH3770 | 35 | 26 |  |  |  |  |
| REN106I07 | 36 | 7 |  |  |  |  |
| REN214H22 | 36 | 23 |  |  |  |  |
| FH3272 | 37 | 5 |  |  |  |  |
| H10101 | 37 | 13 |  |  |  |  |
| REN67C18 | 37 | 22 |  |  |  |  |
| FH2532 | 37 | 25 |  |  |  |  |
| REN164E17 | 38 | 23 |  |  |  |  |
